# Pulmonary Congestion Assessment in Heart Failure: Traditional and New Tools

**DOI:** 10.3390/diagnostics11081306

**Published:** 2021-07-21

**Authors:** Filippo Pirrotta, Benedetto Mazza, Luigi Gennari, Alberto Palazzuoli

**Affiliations:** 1 Department of Medicine, Surgery and Neurosciences, University of Siena, 53100 Siena, Italy; pirrotta@student.unisi.it (F.P.); luigi.gennari@unisi.it (L.G.); 2 Cardiovascular Diseases Unit, Department of Medical Sciences, Le Scotte Hospital, University of Siena, 53100 Siena, Italy; b.mazza@student.unisi.it

**Keywords:** heart failure, congestion, clinical assessment

## Abstract

Congestion related to cardiac pressure and/or volume overload plays a central role in the pathophysiology, presentation, and prognosis of heart failure (HF). Most HF exacerbations are related to a progressive rise in cardiac filling pressures that precipitate pulmonary congestion and symptomatic decompensation. Furthermore, persistent symptoms and signs of congestion at discharge or among outpatients are strong predictors of an adverse outcome. Pulmonary congestion is also one of the most important diagnostic and therapeutic targets in chronic heart failure. The aim of this review is to analyze the importance of clinical, instrumental, and biochemical evaluation of congestion in HF by describing old and new tools. Lung ultrasonography (LUS) is an emerging method to assess pulmonary congestion. Accordingly, we describe the additive prognostic role of chest ultrasound with respect to traditional clinical and X-ray assessment in acute and chronic HF setting.

## 1. Introduction

Congestion occurrence is the primary cause of acute HF (AHF) decompensation, and it is considered the main cause of hospitalization [1]. The clinical assessment evaluating peripheral and central signs of congestion is not accurate enough, and all clinical congestion scores proposed have demonstrated modest accuracy [2,3]. The real challenge is to render an early diagnosis of pulmonary congestion before symptom deterioration in order to reduce the hospitalizations. Traditionally, the most used tools behind clinical evaluation are chest radiography (CRx) and natriuretic peptides (NPs) measurement. However, both methods are poorly available to the general medical practitioner at a patient’s home, and they need access to a laboratory or specific clinical wards. Conversely, chest lung ultrasound (LUS) can be performed using a simple echograph with either linear or convex probe, and it does not require a specific skill. B-line counts represent a simple and reliable method to assess pulmonary congestion and to evaluate effective water retention in the lung. A B-line artefact is defined as a laser-like hyperechogenic reverberation arising from the pleural line up to the screen bottom, moving vertically and in synchrony with lung sliding in a way that is similar to that of a comet [4]. Although the screening is relatively simple, there are different methods and approaches to counting the whole number of comets. Some studies used a detailed methodology evaluating 14 different zones for each hemithorax. Other simpler approaches comprise eight- or four-spaces techniques for each chest side [4]. Currently, there is not a gold standard; the examination is usually performed alongside a traditional echocardiographic exam, and the examination depends on the physician’s or ultrasound technician’s experience and amount of time allocated to spend with each patient. Overall, lung ultrasonography is an emerging tool for evaluating congestion in acute and chronic settings, one that could change the current traditional assessment. 

## 2. Clinical Examination

**Figure 1:**
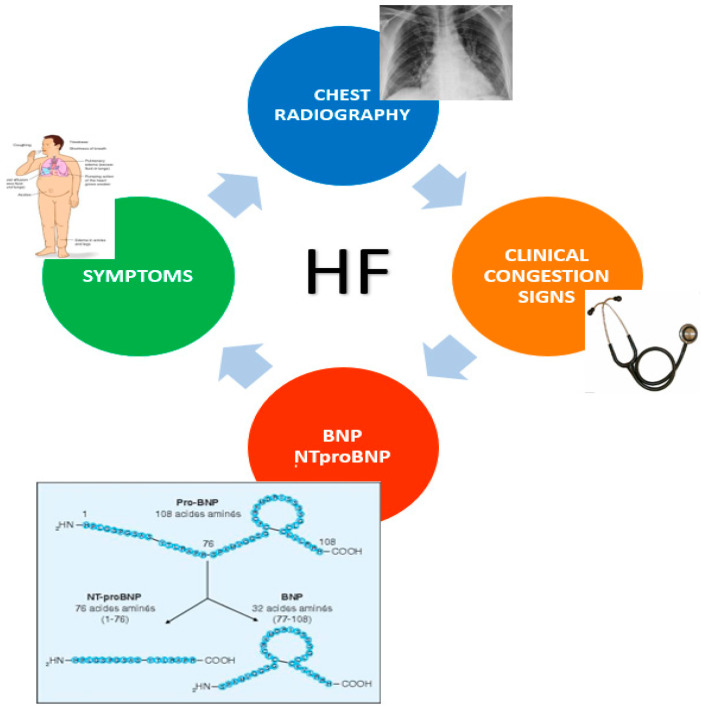
Traditional diagnostic screening for congestion detection in HF: current criteria are based on signs and symptoms, NPs measurement, and chest radiography.

Physical examination is the first step for the detection of congestion and severity. The search for signs and symptoms, such as jugular vein distention, pulmonary crackles, hepatomegaly, dyspnea at rest, presence of peripheral edema, and additional cardiac sound can reveal the presence of central and systemic congestion. Clinical evaluation is important in the identification of pulmonary congestion, but the specificity and sometimes the sensitivity of the signs and symptoms of pulmonary congestion are often scarce [5]. Dyspnea is the principal symptom that can predict the presence of congestion, but it is common among cardiac, respiratory, and systemic diseases. Indeed, its sensitivity and specificity are very low (66% and 52%, respectively). Peripheral edema is a common consequence of hypoproteinemia: malabsorption and hepatic and renal disorders may configure a similar picture due to low albumin levels and oncotic pressure decrease. Some drugs such as dyidropiridine, a calcium channel blocker, or alpha blockers can lead to a bilateral leg edema. Auscultation of rales or crackles may indicate interstitial pulmonary edema, but it is not present during early phases of decompensation and during chronic pulmonary venous congestion. Therefore, other pulmonary diseases such as interstitial fibrosis or pneumonia may include these signs. Crackles could be found in other pulmonary conditions such as chronic bronchitis, asthma, and emphysema, which can affect lungs at the same time [6]. According to previous studies, combining and grading together all signs and symptoms in a Congestion Score algorithm allows a notable improvement of the diagnostic process. Even though clinical symptoms and signs are late manifestations of congestion, clinical examination is a good approach for predicting 6-month event-free survival in patients with acute decompensation [7,8,9]. Among patients developing acute decompensated HF, pulmonary congestion (characterized by interstitial and alveolar edema) precedes clinical symptoms and manifestations of congestion. While clinical congestion may be quickly resolved with treatment, pulmonary congestion may persist for longer, and its resolution during hospitalization may be delayed or incomplete despite aggressive diuretics. Furthermore, decompensation could be clinically silent in some patients with chronic heart failure (CHF), and it could remain unrecognized until the occurrence of relevant symptoms requires hospitalization [10]. For these reasons, a more accurate evaluation through the combination of different imaging methods appears the most reliable solution (Figure 1).

## 3. Chest Radiography

Chest radiography (CRx) is a fast and inexpensive method performed especially in the Emergency Department (ED) as a first-line diagnostic imaging modality in patients with acute dyspnea [11,12,13]. However, its diagnostic accuracy for HF has been reported to be relatively low [14,15]. In particular, diagnosing HF in patients with concomitant lung diseases, such as chronic obstructive pulmonary disease (COPD) and pneumonia, still remains challenging. Moreover, the radiographic resolution of the images in some population, such as patients with obesity or bedridden elderly, is significantly reduced. The role of CRx changes in relation to clinical presentation and timing: in a patient with de novo HF, its accuracy remains high, whereas in those with repetitive episodes and recurrence of CHF, in which a chronic pulmonary vein hypertension has occurred, the diagnostic power is less accurate. Similarly, in AHF and CHF, the diagnostic value is debated. Killip’s classification is one of the most used modalities to assess heart failure severity in symptomatic patients, and it is used as prognostic scale. CRx allows evaluation of only the most severe Killip classes (III, IV, rarely also II). This demonstrates that the isolated approach with CRx in patients with AHF is useful for the evaluation of the most advanced stages, but it does not recognize the earliest classes, resulting in a delayed diagnosis and possible mistreatment. Although it is useful for detecting pulmonary edema, the absence of other radiological evidence in chronic outpatients does not exclude elevated filling pressure [16]. A serial assessment of a large population admitted in the ED with dyspnea was conducted, and 18% had a negative CRx, while approximately 1 of every 5 subjects with AHF did not demonstrate signs of chest congestion [17]. Therefore, serial CRxs are not recommended in the assessment of pulmonary congestion in CHF. Further limitations of CRx are exposure to radiation, operator-dependent quality, interobserver [18,19] variability, and the detection of only the most extreme extravascular pulmonary water variations [18,19,20]. For these reasons, supplementary laboratory and diagnostic tests may be warranted. 

## 4. Natriuretic Peptides

B-type natriuretic peptide (BNP) was initially described by Japanese researchers in 1988. An early study, published in 1994, showed that this biomarker could help distinguish between cardiac and non-cardiac causes of dyspnea. Since the initial studies showing the usefulness of NPs for HF diagnosis, a vast number of other clinical applications for these neurohormones have emerged. NPs are now being used in outpatient heart failure clinics, for screening programs, and for risk-prediction algorithms in various settings. Over last 10 years, NPs measurement significantly increased diagnostic sensitivity, and it reduced the diagnostic time process in patients with dyspnea [21]. For these reasons, American and European guidelines introduced these biomarkers into the diagnostic algorithm of patients presenting with AHF. Because of their diagnostic and prognostic power, NPs assays are now also used in chronic setting to evaluate disease status, congestion degree, and response to therapy [22].

These hormones have a very high negative predictive value that allows dyspnea of cardiogenic origin to be ruled out when values are in the normal range. In particular, their contribution is important in the assessment of new-onset heart failure [23]. NPs are not only important for the diagnostic accuracy of HF, but they also provide relevant prognostic information. An elevated cutoff value of NPs at discharge is one of the best mortality predictors. In hospitalized patients, a reduction in NT-proBNP from treatment entry to discharge of less than 30% has been related to an increased probability of rehospitalization and death [24]. Serial BNP measurement was also demonstrated to be an important marker for risk stratification in patients with CHF [25]. Elevated levels are associated with a higher incidence of recurrent episodes of heart failure or sudden death [26]. Plasma NPs values range according to the type of HF and the presence of associated diseases: among patients with similar NYHA class, hormones are reduced in patients with heart failure and preserved ejection fraction (HFpEF) compared with those with heart failure and reduced ejection fraction (HFrEF), but they remain still higher than in controls [27].

NPs elevations are closely related to high cardiac filling pressure values, parietal stress, and distension, which are the most important triggers for hormone release. Throughton et al. [28] evaluated the changes in serum levels of NPs in stable patients with ventricular systolic dysfunction in relation to some echocardiographic parameters including the degree of diastolic dysfunction, the degree of right ventricular dysfunction, and the severity of mitral regurgitation. BNP values were closely correlated with some indices of diastolic dysfunction, such as deceleration time (DT), E/e’, and the velocity of the transmitral flow—all the parameters that reflect an increase in filling pressures. Serum levels of NPs increased significantly in accordance with the degree of diastolic dysfunction. Furthermore, the increase in NPs is closely correlated with systolic dysfunction, left ventricle filling pressure pulmonary, hypertension, and right ventricular dysfunction [29,30]. The importance of NPs in the detection of pulmonary congestion must be emphasized, particularly in the forms in which the clinical signs of congestion are scarcely recognizable. Several studies showed that enrolled patients with either AHF or CHF had NPs values at admission, despite the symptoms’ absence and negative clinical assessment. Therefore, numerous studies have focused their attention on the correlation between NPs and echocardiography in pulmonary congestion recognition [28,31,32].

Although these blood tests have a very high sensitivity, their specificity remains relatively low. In fact, several features may influence baseline levels: chronic kidney disease, atrial fibrillation, and ischemic heart disease tend to increase the value, whereas other conditions such as obesity and HFpEF showed lower levels. In patients with acute renal failure, NPs levels may be increased independently of the presence of cardiac dysfunction; therefore, diagnosing CHF in this population should rely on more standard criteria. Despite these findings, NT-proBNP has recently been demonstrated to be useful both in diagnosing and excluding CHF across a wide spectrum of renal function, but higher cutoff points may be applied [33].

## 5. Emerging Role of Chest Ultrasound in HF Diagnosis 

Lung ultrasonography (LUS) is a quantitative, simple, economic, and rapid method to assess pulmonary congestion [4]. This diagnostic tool is widely available, particularly in acute settings, while in outpatient primary care it is less practicable due to the limited experience of general doctors and the lack of ultrasounds machines’ distribution. Therefore, LUS requires some experience in an accredited echo lab. The association of this tool with clinical and NPs evaluation has greatly increased the diagnostic accuracy of heart failure. LUS consists of the measurement of discrete laser-like vertical hyperechoic reverberation artifacts arising from the pleural line, extending to the bottom of the screen without fading, and moving synchronously with lung sliding [4]. These artifacts are defined as “B-lines” by scanning along the intercostal spaces using either a phased array or curvilinear transducer.

After critical analysis of previous studies, we can discern between two different methods quantifying B-lines: a score approach and a count-based method. The former method considers a minimum number of B-lines for each space as a “positive” zone (typically at least three B-lines) and then adds up the number of positive zones [34,35]. The latter method consists of counting B-lines in each zone and summarizing all B-lines for each lung [36,37]; when comets are confluent between two different zones, their number can be estimated from the percentage of space they occupy on the screen below the pleural line, divided by 10 (i.e., if about 70% of the screen below the pleural line is occupied by B-lines, it would conventionally count as 7 B-lines, up to a maximum of 10 per zone) [38]. 

The subdivisions of the chest into 8 zones and 28 sub-zones permits the best quantification of B-line count. The eight zones of interest are the anterior and lateral hemi-thoraxes, scanning along the parasternal, midclavicular, anterior axillary, and medium axillary line from the second to the fifth intercostal space on the right hemithorax, and from the second to the fourth intercostal space on the left hemithorax. Each of the mentioned areas are in turn subdivided into four further zones. The subspaces belonging to the fifth left intercostal space are excluded from the count [4]. 

Nevertheless, the role of congestion in diagnosis of HF has been emerging over last 10 years. Different approaches in different populations and settings and the analysis of patients with various clinical characteristics and different congestion status has led to diversity in terms of cutoffs and scan procedures. The first pioneering study analyzing the additive diagnostic role of chest ultrasound in ambulatory outpatients reported a significant correlation among B-line congestion scores and NPs levels. Therefore, a B-line cutoff >15 was described as a reliable threshold for pulmonary congestion. That report was the first to demonstrate that LUS assessment was a simple and relatively accurate method to assess decompensation [39]. Other studies demonstrated that a total amount of ≤5 B-lines was defined as a normal echographic chest pattern, as it has been reported that healthy patients may have a small number of comets, especially confined laterally to the last intercostal spaces above the diaphragm [40]. Despite these findings, another study of 195 ambulatory patients comparing LUS with clinical congestion evaluation found that a number of >3 B-lines was associated with increased risk of hospitalization or death [37]. Therefore 80% of patients in higher LUS terciles did not show any signs of congestion during clinical examination, and only 19% had crackles at auscultation [37]. Current discrepancies confirm that there are not uniform criteria and that better standardization appears desirable in order to provide a consistent message to clinicians.

Since the diagnostic role and exact threshold in outpatients is debated, LUS assessment in acute care settings is probably much more accurate. Indeed, Coiro et al. describe sixty consecutive acute care patients who underwent clinical echocardiographic and LUS examinations, in which B-lines >30 was associated with improved diagnostic accuracy and predicted a combined end point during 3-month follow-up [41].

In a direct comparison between AHF with either reduced or with preserved ejection fraction, we found a similar number of B-lines in both HF subtypes; although it was slightly higher in those with reduced ejection fraction, the correlation with the other echo parameters of congestion remained similar. Therefore, a cutoff value >32 was predictive of poor outcome. Pellicori et al. showed that a cutoff value of ≥3 B-lines in at least two zones per hemithorax (of six to eight evaluated zones in total) had substantial sensitivity (94–97%) and specificity (96–97%) in patients with AHF—higher than in physical examination, CRx, and NT-proBNP) (sensitivity 85%, specificity 89–90%) [42]. Finally, the last meta-analysis comparing LUS with CRx suggests that B-line counts are more sensitive than radiography in detecting pulmonary edema and that it should be included as an additional modality in patients presenting with acute dyspnea [14].

The role of B-lines in guiding therapy is another interesting field because in theory the serial LUS examination during hospitalization could help in treatment tailoring and congestion grading before discharge. The simple difference between B-line counts at admission and before discharge could guide physicians in therapy optimization. Similarly, in a study conducted by Cortellaro et al., LUS analysis was applied for assessing the effective determination of pulmonary congestion and diuretic response after diuretic therapy during the first 24 h after admission. Significant B-line differences between admission and ongoing treatment was associated with good diuretic response and relevant decongestion [43]. As reported in Platz et al., studying the evaluation of the B-lines at 3 h from admission and from the beginning of therapy showed an improvement in pulmonary congestion, intended as a reduction of the B-lines, and this fact provides a real-time approach to therapy, allowing a better titration of therapy that is based on the type of patient and the severity of the acute congestion. Although in this study a cutoff of 3 h was considered, as far as we know from previous studies, there is no exact cutoff time to evaluate the effect of therapy [44]. The ability to perform a rapid evaluation and to compare the first findings with a subsequent scan is one of the main practical advantages of LUS detection in HF (Table 1) [35,38,42,43,44]. 

## 6. Prognostic Role of Chest Ultrasound

A high number of B-lines at the time of discharge from a hospitalization for AHF or in ambulatory patients with CHF identifies those at high risk of subsequent HF readmissions or death in observational studies [42].

Over the years, several studies have been conducted in order to establish the effectiveness of B-lines in the prognostic evaluation of patients with HF. Gustafsson et al., found that a high number of B-lines assessed by a five-zone method adjusted for age, systolic function, and NT-proBNP identified an increased the risk of death or hospitalization after 6-month follow-up [47].

Gargani et al. [40] showed that patients with persistent sonographic pulmonary congestion have an increased risk for rehospitalization for HF in the next 6 months. Patients with ≤15 B-lines before discharge presented a very low risk of rehospitalization. Platz et al. [44] reported that ambulatory HF patients ≥3 B-lines in eight chest zones were at higher risk for HF hospitalization or death over follow-up period of 180 days. Additionally, Coiro et al. showed that in AHF patients LUS examination together with NYHA class and baseline log-BNP was capable of identifying patients with higher risk for rehospitalization [41]. A cutoff ≥30 B-lines at discharge was a very powerful predictor of post-discharge outcome in terms of risk of death, HF hospitalization, and the combined endpoint during 3-month follow-up. Compared with ≥15 B-lines at discharge, Coiro et al. proposed ≥30 B-lines at discharge as the best B-line predictor of outcome, assessed by multivariate analysis [41]. Similarly, Cogliati et al. found that a 1-point increase in the ultrasound score of the B-lines was associated with an approximately 24% adverse event risk increase within 100 days [45]. Current findings highlight the importance of assessing subclinical pulmonary congestion as a potential reason to modify treatment and to reduce the risk of HF hospitalization [40,41,42,44,45].

In our previous study [46], we identified that a cutoff point of >22 B-lines at discharge did not show significant differences between patients with HFrEF and HFpEF. These threshold differences may have been a result of the time frame of the scan and the effective type of HF congestion that appeared during examination and the start of treatment. Therefore, some associated diseases and comorbidities may affect B-line count. We demonstrated that patients with increased body mass index (BMI) experienced a lower cutoff value compared with those with normal weight [46]. Other situations potentially influencing LUS examination are contemporary presence of pneumonia, interstitial lung disease, or cachectic status with low plasma protein level, in which B-line count could be misinterpreted and overestimated. Thus, although the comet count is a good parameter to estimate the congestion situation [39,41,42], LUS must be contextualized in each specific patient situation. According to current assumptions, it would be useful to outline different profiles based on the variations of B-lines in each patient, evaluating the number of comets at admission, during hospitalization, and at discharge. Establishing a precise timing would also allow better standardization of the scores. Interestingly, a low B-line number is relevant in order to rule out HF diagnosis and congestion: a number <5 B-lines can be a good negative predictor of the presence of pulmonary congestion [40]. However, the opposite is not true.

## 7. Relevance of Lung Ultrasound in Different Settings

B-LINES IN PRIMARY CARE—Early diagnosis of HF can reduce the number of hospitalizations with related consequences on quality of life and risk. An early diagnosis could be performed in the setting’s general population by a specific diagnostic screening, including clinical assessment and rapid ultrasound chest and abdominal scan. Indeed, in primary care (PC), HF diagnosis by simple check-up is difficult because of the symptoms’ non-specificity and the late onset of clinical signs of congestion. For this reason, LUS can be an additive, easy, and rapid method for ruling-in patients with a new diagnosis of HF. A recent study evaluating LUS in primary care showed that the evaluation of B-lines in anterior, lateral, and posterior areas demonstrated a positive predictive value (92%) to accurately rule-in HF patients [49]. Associating this value with the negative predictive value of NPs can significantly reduce the diagnostic delay that may lead to treatment delay. Considering that the availability of the NPs assay is not always guaranteed in PC clinics, LUS appears an alternative tool for physicians, and its application may be extended for initial screening of the general population.

B-LINES IN CHF OUTPATIENTS—Since almost 90% of HF recurrence is related to congestion, the presence of pulmonary congestion may identify patients with high risk of HF hospitalization and death [44]. For this reason, lung congestion quantification permits a better stratification of risk in chronic HF patients and can permit the appropriate control and management of CHF patients at follow-up. A recent study including 577 patients showed the importance of B-line count as an independent prognostic factor. Quantifying B-line number in quartiles identifies patients at high risk—in particular, a high number of B-lines identified patients with a 2.6× increased risk of death and rehospitalization [50]. Like PC, outpatients who are followed-up with LUS can achieve a relevant improvement of prognostic stratification by a tailored treatment focused on decongestive therapy optimization that is LUS-guided.

B-LINES AS GUIDE FOR TREATMENT—Treatment with diuretics—in particular, loop diuretics—in patients with HF plays an important role in reducing pulmonary congestion. Because LUS reflects the dynamic variation in pulmonary water content, a serial and repetitive screening could be of relevance in both acute HF and chronic HF patients prone to multiple rehospitalization (Frequent Flyers). Assessment and quantification of pulmonary congestion may help to guide treatment in patients with HF and help to improve their prognosis [51]. Rivas-Lasarte et al. conducted a case-control study, considering 123 patients, divided in a LUS group and a control group. A LUS-guided diuretic therapy titration was performed in the former [52]. Considering hospitalization for worsening HF and all-cause death as primary end points, this study showed a reduced number of decompensations in the LUS group versus an increase of number of urgent visits for those with decompensated HF in control group. Accordingly, another report, found significant improvement of walking capacity observed by the 6 min test in patients monitored by LUS [53]. Diuretic tailoring of dose by a guided-LUS approach can significantly reduce the episodes of HF decompensation, avoiding redundant hospitalization with improvement of quality of life together with diuretic titration optimization.

## 8. Current GAPS and Potential Applications

Despite the elevated sensitivity of B-line findings in pulmonary congestion assessment, B-line number could change when patients assume different positions: the traditional position for LUS acquisition is a semi-supine standing that allows both the posterior and anterior chest zones to be viewed. This is not always possible in patients with acute dyspnea who cannot assume such a forced position. Another weakness is related to the exam specificity: B-line occurrences are not only proper for use in HF, but they are relevant for use in several pulmonary disease, such as pulmonary fibrosis and COPD, acute respiratory distress syndrome (ARDS) and interstitial pneumonia [40]. A recent new application of chest ultrasound scan has been proposed in COVID-19 patients in order recognize the side and extent of pulmonary infection. Some regions adopted the pulmonary scan for SARS-CoV patient screening and hospitalization decisions. Moreover, in hospitalized patients, the chest scan became an additional tool alongside CRx for chest tomography. However, in these patients, B-lines demonstrated specific characteristics; being mainly located in the subpleural area, the diagnostic differentiation of patients with suspected HF is complex, and it requires additional laboratory and imaging tools [54]. Other concerns are related to BMI. Because higher BMI is associated with more thoracic sub-cutaneous adipose tissue and greater distance between the probe and pleural line, the correct B-lines identification during chest examination can be difficult, and it could result in underestimation. Similar to the NPs, B-line counts tend to decline with increasing BMI [48]. A more universal approach should be recommended; indeed, published research has applied various methodologies with different scan windows and chest areas. This differentiation led to a lack of uniformity and results discrepancies, creating confusion for clinicians and resulting in diagnostic algorithms that are not standardized.

**Figure 2:**
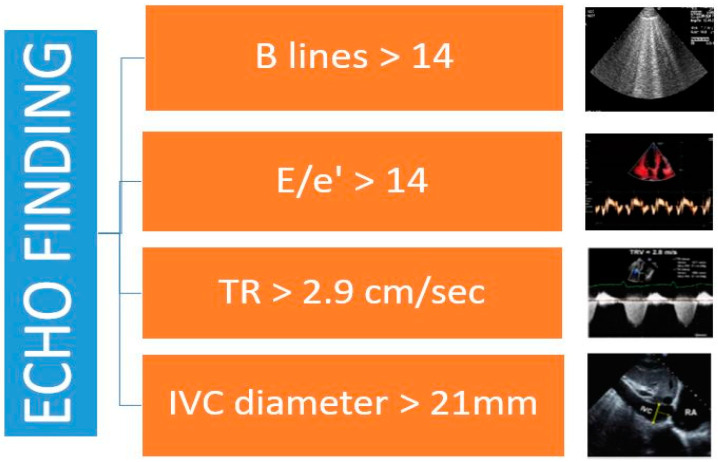
Echographic findings in HF.

Current weakness may be bypassed using an integrated approach that includes different imaging tools. As reported in a recent study, B-line analysis together with BNP and cava vein distention lead to a more accurate diagnosis and better risk stratification [48]. Current ESC guidelines do not recommend the contemporary use of LUS and inferior cava vein (IVC) diameter for the assessment of volume status; conversely, a toolbox for congestion determination should comprise LUS, central congestion evaluation by echocardiographic assessment, and vascular ultrasound analysis. Recently, some papers have suggested the application of a complete ultrasound score, including a detailed analysis of echo parameters for non-invasive estimation: E/e’ is a good mirror of increased left ventricular filling pressure, tricuspid regurgitation is closely related to invasive measurement of systolic pulmonary pressure, integral velocity time of pulmonary flow is related to mean pulmonary artery pressure, and cava vein diameter is a good marker of central vein pressure [55,56]. Thus, the contemporary measurement of all these variables may better define the effective central and peripheral congestion for each patient affected by HF (Figure 2) [34]. 

Although this diagnostic algorithm may reduce misdiagnosis and the number of false positives, it is only partially applicable in the emergency setting and in ambulatory patients because it requires a specific experience and cardiology competence.

## 9. Conclusions

Clinical congestion plays a central role in HF exacerbation and in its detection during early-phase avoidance of recurrent hospitalization and functional deterioration. Thus, congestion detection before HF-related symptom occurrence remains a goal for clinical management. Typically, clinical assessment mainly reveals peripheral water retention and increased central venous signs, but it is less specific in the detection of pulmonary congestion. Similarly, the traditional CRx has good accuracy in AHF and in more advanced stages, but it is less accurate in chronic settings. In this framework, LUS demonstrated optimal diagnostic power and good prognostic relevance. Some gaps remain with regard to the different methods and applications as well as to the diagnostic cutoff capable of ruling-in an HF diagnosis. The contemporary assessment of some echocardiographic findings of congestion with B-line assessment may facilitate HF diagnosis and monitoring. The future challenge is to recognize the best available imaging variables of congestion and to apply a standardized algorithm in clinical practice.

## Figures and Tables

**Table 1:** Diagnostic accuracy of different LUS analyses in pulmonary congestion evaluations compared with clinical congestion evaluation NPs measurements and relationship with echocardiographic parameters.

			**AHF**			
**Author of the study**	**N. patients**	**B-lines number/score**	**Congestion signs, *n* (%)**	**NT-proBNP/** **BNP (pg/mL)**	**Eco parameters admission**	**Accuracy**
Coiro et al. (2015) [41]	60	Assessed on 8 and 28 chest zones. Method: score and count. Score ≥ 3 zone: positive zones. Count: sum of B-lines in all zones > 30	Crackles: 18 (30%); Leg edema: 11 (18%)	BNP: 575 (228.5–1147)	E/E′: 19.11 ± 9.5; IVC diameter (mm): 19.71 ± 5.16; TAPSE (mm): 17.26 ± 3.8; EF (%): 37.5% ± 15	Outcome: composite: 3-month HF hospitalization or death. ≥30 B-lines (HR 5.66, 95% Cl 1.74–18.39, *p =* 0.04)
Gargani et al. (2015) [40]	100	Assessed on 28 chest zones. Method: score and count. Score: mild: 6–15 B-lines; moderate: 16–30 B-lines; severe: > 30 B-lines. Count: sum of B-lines in all zones	Not assessed	NT-proBNP: 5291 ± 5877	Pseudonormal pattern 11%; Restrictive pattern 23%; PAPs (mmHg): 49 ± 15; TAPSE (mm): 16.5 ± 4.7	Outcome: composite HF hospitalization or death (mean follow-up 159 days). >15 B-lines- readmission for HF. Sens: 100% spec: 64.8%. >20 B-lines- readmission for HF. Sens: 100% spec: 74.7% NPV: 100%
Cogliati et al. (2016) [45]	149	Assessed on 8 and 28 chest zones. Method: Score (8 zones) Count (28 zones). Score: ≥3 B-lines per zone: positive zone. Count: sum of B-lines in all zones. Count: total B-lines > 15; Total B-lines > 30	Peripheral edema, JVT (jugular vein turgescence) and pulmonary crackles.	NT-proBNP: 2407 ± 1400	E/E′ > 15. 41.6% pz (49) TAPSE (mm): 16.5	Outcome: composite 100-day HF hospitalization or all-cause death. Correlation between the sonographic score and event occurrence (HR 1.19; CI 1.05 to 1.34; *p* = 0.005) increase of 24% the risk of outcome.
Cortellaro et al. (2016) [43]	41	Assessed on 11 chest zones. Method: Score. <3 B-lines in a zone (0 points); ≥3 B-lines in ≥1 zone (1 point); multiple/confluent B-lines (2 points)	Dyspnea, orthopnea, paroxysmal nocturnal dyspnea, rales	NTproBNP: 5867 ± 6112	After therapy: IVC diameter (cm): 1.9 ± 0.5 TAPSE (mm) 20.0 ± 3.5 PAPs (mmHg) 37.2 ± 9.8 E/E′: 14.8 ± 5.2	Mean B-score significantly decreased at T3 (from 1.59 ± 0.40 to 0.73 ± 0.44, *p* < 0.001) and between T3 and T 24 (from 0.73 ± 0.44 to 0.38 ± 0.33, *p* < 0.001).
Palazzuoli et al. (2018) [46]	162	B-line count > 22	Crackles: 140 (86%) Hepatomegaly: 56 (35%) 3th tone: 48 (41%) JVD: 68 (42%) Leg edema: 102 (63%)	BNP HFrEF: 1164 ± 420 BNP HfpEF: 889 ± 130	E/e′: 16 ± 4 TAPSE (mm) 20 ± 4	Sens: 70%; spec: 81%; accuracy: 76%
**CHF**						
**Author of the study**	**N. patients**	**B-lines number/score**	**Congestion signs, *n* (%)**	**NT-proBNP/** **BNP (pg/mL)**	**Eco parameters admission**	**Accuracy**
Miglioranza et al. (2013) [39]	97	Score ≥ 3 zone: positive zone > 15	CCS > 2: 44 (66.7%) s3–s4: 7 (7%)	NT-proBNP: 3070 ± 3100	E/e′: 23 ± 16; PAPm: 39.1 ± 10.9;	Combining NT-proBNP > 1000 pg/mL and/or E/e′ > 15: yielded a C-statistic of 0.89 for LUS spec: 83.3%; sens: 84.9%; AUC delta: 0.194, 95% CI 0.147, 0.315; *p =* 0.001 primary outcome
Platz et al. (2016) [37]	185	Assessed on 8 chest zones. Method: count. Sum of B-lines in all zones Score ≥ 3 zone: positive zone > 15	Crackles: 35 (19%) JVD: 68 (37%) Leg edema: 65 (35%)	NT-proBNP: 5086 (3023–9248)	EF < or ≥ 45%	Outcome: composite: 6-month HF hospitalization or all-cause mortality. AUC delta: 0.132, 95% CI 0.078, 0.213; *p* < 0.001)
Gustafsson et al. (2015) [47]	104	Assessed on 5 chest zones. Method: count and score. Score: ≥ 3 B-lines in one zone. Count sum of B-lines in all zones.	Not assessed	NT-proBNP: 1820 ± 1000	IVC diameter (mm): 18 ± 4	Outcome: composite 6-month HF hospitalization or all-cause mortality. B-lines cox proportional HR adjusted for age > 72, NT-proBNP and LV function. B-lines > 3 (HR 2.9 (1.3–6.6), *p* = 0.11, HR 3.5 (1.5–7.9), 0.003; Age > 72 HR 0.3 (0.2–0.8), *p =* 14; EF <40% HR 0.7 (0.3–1.6) *p* = 0.70; NT-proBNP HR 3.5 (1.5–8.5) *p = 0*.005
Pellicori et al. (2019) [48]	342	Assessed on 28 chest zones. Method: count. B-lines (≥14)	Crackles: 40 (11.7%) JVP: 55 (16.1%) peripheral edema: 103 (30.1%) No signs of congestion: 205 (59.9%)	NT-ProBNP HFrEF: 1494 (684–3502) HFmrEF: 1330 (382–2881) HFpEF: 1100 (354–1994)	JVD RATIO mediana: 5.3 TAPSE (mm) mediana: 19 E/e′ lat: mediana: 11 IVC diameter (cm): 2.0	Outcome: (Composite of all-cause mortality or heart failure hospitalization). B-lines-HR: 1.02 (1.01–1.03); Chi2: 26.3; *p* < 0.001

## Data Availability

Not applicable.

## Abbreviations

AHF	Acute Heart Failure
ARDS	Acute Respiratory Distress Syndrome
BNP	Brain Natriuretic Peptide
CHF	Chronic Heart Failure
COPD	Chronic Obstructive Pulmonary Disease
CRX	Chest X-ray
HF	Heart Failure
HFpEF	Heart Failure with Preserved Ejection Fraction
HFrEF	Heart Failure with Reduced Ejection Fraction
IVC	Inferior Cava Vein (IVC)
LUS	Lung Ultrasounds
NPs	Natriuretics Peptides

## References

[1] Mentz R.J., Stevens S.R., DeVore A.D., Lala A., Vader J.M., AbouEzzeddine O.F., Khazanie P., Redfield M.M., Stevenson L.W., O’Connor C.M. (2015). Decongestion strategies and renin–angiotensin–aldosterone system activation in acute heart failure. JACC Heart Fail..

[2] Lok C., Morgan C.D., Ranganathan N. (1998). The Accuracy and Interobserver Agreement in Detecting the ‘Gallop Sounds’ by Cardiac Auscultation. Chest.

[3] Ambrosy A.P., Pang P., Khan S., Konstam M.A., Fonarow G., Traver B., Maggioni A.P., Cook T., Swedberg K., Burnett J.C. (2013). Clinical course and predictive value of congestion during hospitalization in patients admitted for worsening signs and symptoms of heart failure with reduced ejection fraction: Findings from the EVEREST trial. Eur. Heart J..

[4] Volpicelli G., Elbarbary M., Blaivas M., Lichtenstein D.A., Mathis G., Kirkpatrick A.W., Melniker L., Gargani L., Noble V.E., International Liaison Committee on Lung Ultrasound (ILC-LUS) for the International Consensus Conference on Lung Ultrasound (ICC-LUS) (2012). International evidence-based recommendations for point-of-care lung ultrasound. Intensiv. Care Med..

[5] Remes J., Mlettinen H., Reunanen A., Pyörälä K. (1991). Validity of clinical diagnosis of heart failure in primary health care. Eur. Heart J..

[6] Gheorghiade M., Follath F., Ponikowski P., Barsuk J., Blair J.E., Cleland J.G., Dickstein K., Drazner M.H., Fonarow G., Jaarsma T. (2010). Assessing and grading congestion in acute heart failure: A scientific statement from the Acute Heart Failure Committee of the Heart Failure Association of the European Society of Cardiology and endorsed by the European Society of Intensive Care Medicine. Eur. J. Heart Fail..

[7] Beck da Silva L., Mielniczuk L., Laberge M., Anselm A., Fraser M., Williams K., Haddad H. (2004). Persistent orthopnea and the prognosis of patients in the heart failure clinic. Congest. Heart Fail..

[8] Breidthardt T., Moreno-Weidmann Z., Uthoff H., Sabti Z., Aeppli S., Puelacher C., Stallone F., Twerenbold R., Wildi K., Kozhuharov N. (2018). How accurate is clinical assessment of neck veins in the estimation of central venous pressure in acute heart failure? Insights from a prospective study. Eur. J. Heart Fail..

[9] Rohde L.E., da Silva L.B., Goldraich L., Grazziotin T.C., Palombini D.V., Polanczyk C.A., Clausell N. (2004). Reliability and prognostic value of traditional signs and symptoms in outpatients with congestive heart failure. Can. J. Cardiol..

[10] Gheorghiade M., Filippatos G., De Luca L., Burnett J. (2006). Congestion in Acute Heart Failure Syndromes: An Essential Target of Evaluation and Treatment. Am. J. Med..

[11] Girerd N., Seronde M.F., Coiro S., Chouihed T., Bilbault P., Braun F., Kenizou D., Maillier B., Nazeyrollas P., Roul G. (2018). Integrative Assessment of Congestion in Heart Failure Throughout the Patient Journey. JACC Heart Fail..

[12] Chouihed T., Manzo-Silberman S., Peschanski N., Charpentier S., Elbaz M., Savary D., Bonnefoy-Cudraz E., Laribi S., Henry P., Girerd N. (2016). Management of suspected acute heart failure dyspnea in the emergency department: Results from the French prospective multicenter DeFSSICA survey. Scand. J. Trauma Resusc. Emerg. Med..

[13] Ponikowski P., Voors A.A., Anker S.D., Bueno H., Cleland J.G., Coats A.J., Falk V., González-Juanatey J.R., Harjola V.P., Jankowska E.A. (2016). 2016 ESC Guidelines for the diagnosis and treatment of acute and chronic heart failure: The Task Force for the diagnosis and treatment of acute and chronic heart failure of the European Society of Cardiology (ESC). Developed with the special contribution of the Heart Failure Association (HFA) of the ESC. Eur J. Heart Fail..

[14] Maw A.M., Hassanin A., Ho P.M., McInnes M.D.F., Moss A., Juarez-Colunga E., Soni N.J., Miglioranza M.H., Platz E., DeSanto K. (2019). Diagnostic accuracy of point-of-care lung ultrasonography and chest radiography in adults with symptoms suggestive of acute decompensated heart failure: A systematic review and meta-analysis. JAMA Netw. Open.

[15] Mueller-Lenke N., Rudez J., Staub D., Laule-Kilian K., Klima T., Perruchoud A.P., Mueller C. (2006). Use of chest radiography in the emergency diagnosis of acute congestive heart failure. Heart.

[16] Chakko S., Woska D., Martinez H., de Marchena E., Futterman L., Kessler K.M., Myerberg R.J. (1991). Clinical, radiographic, and hemodynamic correlations in chronic congestive heart failure: Conflicting results may lead to inappropriate care. Am. J. Med..

[17] Collins S.P., Lindsell C., Storrow A.B., Abraham W.T. (2006). Prevalence of Negative Chest Radiography Results in the Emergency Department Patient with Decompensated Heart Failure. Ann. Emerg. Med..

[18] Picano E. (2004). Sustainability of medical imaging. BMJ.

[19] Nieminen M.S., Bohm M., Cowie M.R., Drexler H., Filippatos G.S., Jondeau G., Hasin Y., Lopez-Sendon J., Mebazaa A., Metra M. (2005). Executive summary of the guidelines on the diagnosis and treatment of acute heart failure: The taskforce on acute heart failure of the European Society of Cardiology. Eur. Heart J..

[20] Hunt S.A., Abraham W.T., Chin M.H., Feldman A.M., Francis G.S., Ganiats T.G., Jessup M., Konstam M.A., Mancini D.M., Keith K. (2005). ACC/AHA 2005 guideline update for the diagnosis and management of chronic heart failure in the adult. A report of the American College of Cardiology/American heart association task force on practice guidelines (Writing Committee to update the 2001 guidelines for the evaluation and management of heart failure). J. Am. Coll. Cardiol..

[21] Alan S., Maisel L., Daniels B. (2012). Breathing Not Properly 10 Years Later: What We Have Learned and What We Still Need to Learn. J. Am. Coll. Cardiol..

[22] Silver M.A., Maisel A., Yancy C.W., McCullough P.A., Burnett J.C., Francis G.S., Mehra M.R., Peacock W.F., Fonarow G., Gibler W.B. (2004). BNP Consensus Panel 2004: A clinical approach for the diagnostic, prognostic, screening, treatment monitoring, and therapeutic roles of natriuretic peptides in cardiovascular diseases. Congest Heart Fail..

[23] Palmer S.C., Yandle T., Nicholls M.G., Frampton C.M., Richards A.M. (2009). Regional clearance of amino-terminal pro-brain natriuretic peptide from human plasma. Eur. J. Heart Fail..

[24] Chow S.L., Alan S.M., Inder A., Biykem B., de Boer A., Felker M., Fonarow G., Greenberg B., Januzzi J., Kiernan M.S. (2017). Role of Biomarkers for the Prevention, Assessment, and Management of Heart Failure. A Scientific Statement from the American Heart Association. Circulation.

[25] Koglin J., Pehlivanli S., Schwaiblmair M., Vogeser M., Cremer P., Vonscheidt W. (2001). Role of brain natriuretic peptide in risk stratification of patients with congestive heart failure. J. Am. Coll. Cardiol..

[26] Berger R., Huelsman M., Strecker K., Bojic A., Moser P., Stanek B., Pacher R. (2002). B-Type Natriuretic Peptide Predicts Sudden Death in Patients with Chronic Heart Failure. Circulation.

[27] Mueller C., Scholer A.A., Laule-Kilian K., Martina B., Schindler C., Buser P., Pfisterer M., Perruchoud A.P. (2004). Use of B-Type Natriuretic Peptide in the Evaluation and Management of Acute Dyspnea. N. Engl. J. Med..

[28] Troughton R.W., Richards A.M. (2009). B-type natriuretic peptides and echocardiographic measures of cardiac structure and function. JACC Cardiovasc. Imaging.

[29] Troisi F., Greco S., Brunetti N.D., Di Biase M. (2008). Right heart dysfunction assessed with echography, B-type natriuretic peptide and cardiopulmonary test in patients with chronic heart failure. J. Cardiovasc. Med..

[30] Goto K., Arai M., Watanabe A., Hasegawa A., Nakano A., Kurabayashi M. (2010). Utility of echocardiography versus BNP level for the prediction of pulmonary arterial pressure in patients with pulmonary arterial hypertension. Int. Heart J..

[31] Palazzuoli A., Beltrami M., Ruocco G., Franci B., Campagna M.S., Nuti R. (2016). Diagnostic utility of contemporary echo and BNP assessment in patients with acute heart failure during early hospitalization. Eur. J. Intern. Med..

[32] Dokainish H., Zoghbi W.A., Lakkis N., Al-Bakshy F., Dhir M., Quinones M., Nagueh S. (2004). Optimal Noninvasive Assessment of Left. Ventricular Filling Pressures A Comparison of Tissue Doppler Echocardiography and B-Type Natriuretic Peptide in Patients with Pulmonary Artery Catheters. Circulation.

[33] Anwaruddin S., Lloyd-Jones D.M., Baggish A., Chen A., Krauser D., Tung R., Chae C., Januzzi J.L.J. (2006). Renal function, congestive heart failure, and amino-terminal pro-brain natriuretic peptide measurement: Results from the Pro-BNP Investigation of Dyspnea in the Emergency Department (PRIDE) Study. J. Am. Coll. Cardiol..

[34] Pivetta E., Baldassa F., Masellis S., Bovaro F., Lupia E., Maule M.M. (2018). Sources of Variability in the Detection of B-Lines, Using Lung Ultrasound. Ultrasound Med. Biol..

[35] Pivetta E., Goffi A., Lupia E., Tizzani M., Porrino G., Ferreri E., Volpicelli G., Balzaretti P., Banderali A., Iacobucci A. (2015). Lung Ultrasound-Implemented Diagnosis of Acute Decompensated Heart Failure in the ED: A SIMEU Multicenter Study. Chest.

[36] Platz E., Campbell R.T., Claggett B., Lewis E.F., Groarke J.D., Docherty K.F., Lee M.M.Y., Merz A.A., Silverman M., Swamy V. (2019). Lung Ultrasound in Acute Heart Failure: Prevalence of Pulmonary Congestion and Short- and Long-Term Outcomes. JACC Heart Fail..

[37] Platz E., Lewis E.F., Uno H., Peck J., Pivetta E., Merz A., Hempel D., Wilson C., Frasure S.E., Jhund P. (2016). Detection and prognostic value of pulmonary congestion by lung ultrasound in ambulatory heart failure patients. Eur. Heart J..

[38] Gargani L. (2019). Ultrasound of the Lungs: More than a Room with a View. Heart Fail. Clin..

[39] Miglioranza M.H., Gargani L., Sant’Anna R.T., Rover M.M., Martins V.M., Mantovani A., Weber C., Moraes M.A., Feldman C.J., Kalil R.A. (2013). Lung ultrasound for the evaluation of pulmonary congestion in outpatients: A comparison with clinical assessment, natriuretic peptides, and echocardiography. JACC Cardiovasc. Imaging.

[40] Gargani L., Pang P., Frassi F., Miglioranza M., Dini F.L., Landi P., Picano E. (2015). Persistent pulmonary congestion before discharge predicts rehospitalization in heart failure: A lung ultrasound study. Cardiovasc. Ultrasound.

[41] Coiro S., Rossignol P., Ambrosio G., Carluccio E., Alunni G., Murrone A., Tritto I., Zannad F., Girerd N. (2015). Prognostic value of residual pulmonary congestion at discharge assessed by lung ultrasound imaging in heart failure. Eur. J. Heart Fail..

[42] Pellicori P., Platz E., Dauw J., ter Maaten J.M., Martens P., Pivetta E., Cleland J.G., McMurray J.J., Mullens W., Solomon S.D. (2020). Ultrasound imaging of congestion in heart failure: Examinations beyond the heart. Eur. J. Heart Fail..

[43] Cortellaro F., Ceriani E., Spinelli M., Campanella C., Bossi I., Coen D., Casazza G., Cogliati C. (2016). Lung ultrasound for monitoring cardiogenic pulmonary edema. Intern. Emerg. Med..

[44] Platz E., Merz A., Jhund P., Vazir A., Campbell R., Mcmurray J. (2017). Dynamic changes and prognostic value of pulmonary congestion by lung ultrasound in acute and chronic heart failure: A systematic review. Eur. J. Heart Fail..

[45] Cogliati C., Casazza G., Ceriani E., Torzillo D., Furlotti S., Bossi I., Vago T., Costantino G., Montano N. (2016). Lung ultrasound and short-term prognosis in heart failure patients. Int. J. Cardiol..

[46] Palazzuoli A., Ruocco G., Beltrami M., Nuti R., Cleland J.G. (2018). Combined use of lung ultrasound, B-type natriuretic peptide, and echocardiography for outcome prediction in patients with acute HFrEF and HFpEF. Clin. Res. Cardiol..

[47] Gustafsson M., Alehagen U., Johansson P. (2015). Imaging Congestion with a Pocket Ultrasound Device: Prognostic Implications in Patients with Chronic Heart Failure. J. Card. Fail..

[48] Pellicori P., Shah P., Cuthbert J., Urbinati A., Zhang J., Kallvikbacka-Bennett A., Clark A.L., Cleland J.G. (2019). Prevalence, pattern and clinical relevance of ultrasound indices of congestion in outpatients with heart failure. Eur. J. Heart Fail..

[49] Conangla L., Domingo M., Lupón J., Wilke A., Juncà G., Tejedor X., Volpicelli G., Evangelista L., Pera G., Toran P. (2020). Lung Ultrasound for Heart Failure Diagnosis in Primary Care. J. Card. Fail..

[50] Domingo M., Conangla L., Lupón J., De Antonio M., Moliner P., Santiago-Vacas E., Codina P., Zamora E., Cediel G., González B. (2020). Prognostic value of lung ultrasound in chronic stable ambulatory heart failure patients. Rev. Esp. Cardiol..

[51] Hman J., Harjola V.P., Karjalainen P., Lassus J. (2018). Focused echocardiography and lung ultrasound protocol for guiding treatment in acute heart failure. ESC Heart Fail..

[52] Rivas-Lasarte M., Alvarez-Garcia J., Fernández-Martínez J., Maestro A., López-López L., Gonzalez E.S., Pirla M.J., Mesado N., Mirabet S., Fluvià P. (2019). Lung ultrasound-guided treatment in ambulatory patients with heart failure: A randomized controlled clinical trial (LUS-HF study). Eur. J. Heart Fail..

[53] Lomoro P., Verde F., Zerboni F., Simonetti I., Borghi C., Fachinetti C., Natalizi A., Martegani A. (2020). COVID-19 pneumonia manifestations at the admission on chest ultrasound, radiographs, and CT: Single-center study and comprehensive radiologic literature review. Eur. J. Radiol. Open.

[54] Palazzuoli A., Ruocco G., Franci B., Evangelista I., Lucani B., Nuti R., Pellicori P. (2020). Ultrasound indices of congestion in patients with acute heart failure according to body mass index. Clin. Res. Cardiol..

[55] Carluccio E., Dini F.L., Biagioli P., Lauciello R., Simioniuc A., Zuchi C., Alunni G., Reboldi G., Marzilli M., Ambrosio G. (2013). The ‘Echo Heart Failure Score’: An echocardiographic risk prediction score of mortality in systolic heart failure. Eur. J. Heart Fail..

[56] Bonios M.J., Kyrzopoulos S., Tsiapras D., Adamopoulos S.N. (2019). Ultrasound guidance for volume management in patients with heart failure. Heart Fail. Rev..

